# Screening and detection of human papillomavirus (HPV) high-risk strains HPV16 and HPV18 in saliva samples from subjects under 18 years old in Nevada: a pilot study

**DOI:** 10.1186/1472-6831-12-43

**Published:** 2012-10-22

**Authors:** Colton Flake, Jamal Arafa, Alex Hall, Eryn Ence, Katherine Howard, Karl Kingsley

**Affiliations:** 1 Department of Advanced Education in Pediatric Dentistry, University of Nevada, Las Vegas – School of Dental Medicine, Las Vegas, Nevada, USA; 2 Department of Environmental and Occupational Health, University of Nevada, Las Vegas – School of Community Health Sciences, Las Vegas, Nevada, USA; 3 University of Nevada, Las Vegas – School of Life Sciences, Las Vegas, Nevada, USA; 4 Department of Biomedical Sciences, University of Nevada, Las Vegas – School of Dental Medicine, Las Vegas, Nevada, USA

**Keywords:** Human papillomavirus, Saliva, Oral screening

## Abstract

**Background:**

Human papillomaviruses (HPV) are oncogenic and mainly associated with cervical cancers. Recent evidence has demonstrated HPV infection in other tissues, including oral epithelia and mucosa. Although a recent pilot study provided new information about oral HPV status in healthy adults from Nevada, no information was obtained about oral HPV prevalence among children or teenagers, therefore, the goal of this study is to provide more detailed information about oral prevalence of high-risk HPV among children and teenagers in Nevada.

**Methods:**

This retrospective study utilized previously collected saliva samples, obtained from pediatric dental clinic patients (aged 2 – 11) and local school district teenagers (aged 12-17) for high-risk HPV screening (n=118) using qPCR for quantification and confirmation of analytical sensitivity and specificity.

**Results:**

A small subset of saliva samples were found to harbor high-risk HPV16 (n=2) and HPV18 (n=1), representing a 2.5% of the total. All three were obtained from teenage males, and two of these three samples were from White participants.

**Conclusions:**

Although this retrospective study could not provide correlations with behavioral or socioeconomic data, this project successfully screened more than one hundred saliva samples for high-risk HPV, confirming both HPV16 and HPV18 strains were present in a small subset. With increasing evidence of oral HPV infection in children, this study provides critical information of significant value to other dental, medical, oral and public health professionals who seek to further an understanding of oral health and disease risk in pediatric populations.

## Background

The human papillomaviruses (HPV) encompass a closely related family of DNA viruses, which are capable of integrating into the human genome to drive transformation of infected epithelia [1-4]. Much of the epidemiological evidence for HPV-driven carcinogenesis, as well as the biological mechanisms, have been derived from studies of cervical cancers [5-7] that isolated high-risk HPV from both adeno- and squamous cell carcinomas [6-9]. Although high-risk HPV drives the transformation and malignancy process of nearly all cervical cancers, HPV infection is now known to modulate epithelial transformation in breast, lung, penile, anal, and also oral tissues [10-20].

The primary risk factors for oral carcinogenesis are tobacco and alcohol use, although new lines of evidence now suggest HPV may also be an independent risk factor [17-23]. The higher prevalence of high-risk HPV strains in pre-cancerous and cancerous oropharyngeal tumors suggests that HPV may preferentially infect developing or established cancers, thereby modulating carcinogenic progression and ultimately influencing health outcomes [24-26]. In fact, some evidence has suggested that high-risk HPV infection may be associated with improved response to treatment and higher survival rates [27-29], while conflicting reports have demonstrated significantly decreased patient survival [30-32] or have observed no discernable and statistically significant effects [33].

Despite the conflicting nature of these results, it is clear that HPV may be involved in modulating tumor responsiveness and carcinogenic progression, therefore many of these studies have provided valuable epidemiological information regarding which high-risk HPV strains are most often implicated in these cases [34-36]. These studies have revealed HPV16, and HPV18 to a lesser extent, accounted for the overwhelming majority (71-94.7%) of high-risk oral HPV detected [17,18,21-23,34-36]. Moreover, new evidence now suggests that these specific high-risk HPV strains (HPV16 and HPV18), may initiate oral carcinogenesis among the smaller fraction of oral cancer patients who do not consume alcohol or use tobacco [37,38].

Based upon the findings of oral HPV in non-tobacco and non-alcohol associated oral cancers, other recent efforts have focused more specifically to evaluate oral HPV prevalence and transmission within healthy populations, which also confirmed that HPV16 and HPV18 were the most commonly detected oral high-risk HPV strains [39-41] among healthy adults.

To this end, a recent pilot study evaluating oral HPV among healthy adults was performed in Nevada [42], a state recently documented with rising rates of oropharyngeal cancers, in stark contrast to the declining rates observed in neighboring states and the US more generally [43,44]. This study revealed the presence of high-risk strain HPV16, but not HPV18, among females and minorities, the only population subgroups demonstrated to have rising orpharyngeal cancer rates – despite the overall declining rates observed within the general population [45-47]. This study utilized a saliva-based screening procedure, part of a growing trend towards less invasive methodologies, such as oral lavage- and saliva-based screenings, to analyze oral HPV status among healthy adult patients [48-51].

Although prevalence rates ranged widely, these studies have suggested that oral HPV infection may be increasing, not only among adults, but more specifically among younger adults, teenagers, and children [52-54].To this end, some studies have examined oral HPV in children or adolescents, although many of these focused primarily on children with underlying medical conditions, such as co-infection with human immunodeficiency virus (HIV) [55-57]. Most other research, however, has focused more specifically on elucidating the role of vertical HPV transmission in newborns, demonstrating the potential to acquire high-risk HPV during the delivery and birthing process, although these infections typically resolve [58-63].

However, new evidence is emerging that demonstrated high-risk oral HPV infection in normal, healthy children, with the highest rates observed among children under 7 years old (7.9%-8.7%), and declining rates observed among healthy adolescents (13-20 years old; 5.1%-5.2%) and healthy adults (3.5%) [64,65]. These observations may suggest that oral HPV infection may occur through close personal contact with family members or through contact with fomites and other vectors at daycare centers, preschool or in primary education settings, with most children immunologically competent to resolve these infections [66]. However, some infections may persist and their contribution to the development of oral cancers and other pathologies remains unclear.

Although a pilot study was recently conducted to evaluate oral HPV status, this involved only healthy adult patients - with no information obtained about oral HPV prevalence among children or teenagers. Based upon the previous evidence demonstrating some level of oral HPV infection in healthy children and adolescents, combined with the lack of data about this population more specifically, the goal of this current study is to provide more detailed information about prevalence of high-risk HPV strains HPV16 and HPV18 in the oral cavity of children and teenagers in Nevada.

## Results

The proportion of female and male specimens from the study sample was not statistically different from the overall proportions within the local community of Clark County, Nevada (Table 1). More specifically, the percentage of females and males in the sample was approximately equal (51.7% and 48.3%, respectively), which was not significantly different than the local area population (*p* = 0.7051). The proportion of minority (non-White) samples was much greater in the study sample (70.3%) than in the local population (39.1%), which was statistically significant (*p* < 0.0001). Although data from the local population were unavailable for age-specific comparisons, the study sample was comprised more from younger children (ages 2-11: 59.3%) than from adolescents and teenagers (12-17: 40.7%).

**Table 1:** Demographic analysis of study participants

	**CCSD (n=48)**	**UNLV-SDM (n=70)**	**Total sample (n=118)**	**Clark County population**	**Statistical analysis**
*Gender*					
Female	n = 30 (62.5%)	n = 31 (44.3%)	n = 61 (51.7%)	n = 969,781 (49.7%)	χ^2^ = 0.17849 d.f. = 1
Male	n = 18 (37.5%)	n = 39 (55.7%)	n = 57 (48.3%)	n = 981,488 (50.3%)	*p* = 0.7051
*Race*					
White	n = 12 (25%)	n = 23 (32.9%)	n = 35 (29.7%)	n = 1,188,323 (60.9%)	χ^2^ = 28.3634 d.f. = 1
Non-White	n = 36 (75%)	n = 47 (67.1%)	n = 83 (70.3%)	n = 762,946 (39.1%)	*p* < 0.0001
*Age*					
2-11	n = 0 (0%)	n = 70 (100%)	n = 70 (59.3%)	N/A	
12-17	n = 48 (100%)	n = 0 (0%)	n = 48 (40.7%)	N/A	

**Figure 1:**
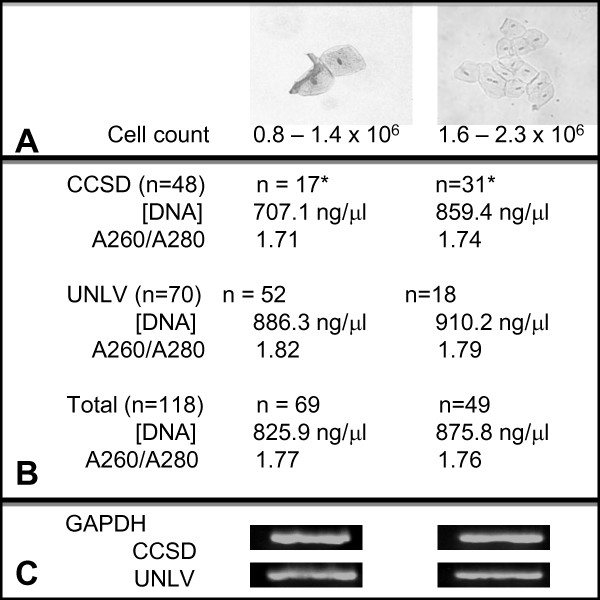
**Saliva sample analysis: cell count, DNA isolation and purity**. **A**) Following centrifugation and cell resuspension in saline, cell number for each sample was determined revealing three-fourths of the UNLV samples were observed to have lower cell counts (0.8-1.4 x 10^6^ cells/mL) while only one-third of CCSD sample were estimated* to have lower cell counts. **B**) DNA concentrations were found to be higher from the high-cell count samples within each cohort (CCSD: 859.4 and 707.1 ng/μL, respectively; UNLV: 886.3 and 910.2 ng/μL, respectively). DNA purity averaged between 1.71 and 1.82. **C**) Equal concentrations of extracted DNA (1 μL) was screened using GAPDH, which revealed no considerable variation in band intensity between cohorts of samples from high- and low-cell count categories.

Detailed analysis of saliva samples revealed some variability between cell counts, DNA concentration and DNA purity between samples (Figure 1). Cell counts varied between a 0.8 – 2.3 x 10^6^ cell/mL, which were further categorized into low (0.8 – 1.4 x 10^6^) and high (1.6 – 2.3 x 10^6^) cell counts. DNA was successfully isolated from all saliva samples, with the average DNA concentration for samples observed at 842.7 ng/μL. Samples from each cohort (CCSD, UNLV) with cell counts in the low category were found to have lower average concentrations of DNA than samples from the same cohort with cell counts in the high category. Absorbance measurements and A260/A280 ratio analysis confirmed the purity of DNA isolates, which was approximately equal between cohorts, as well as between samples in the low- and high-cell count categories.

**Figure 2:**
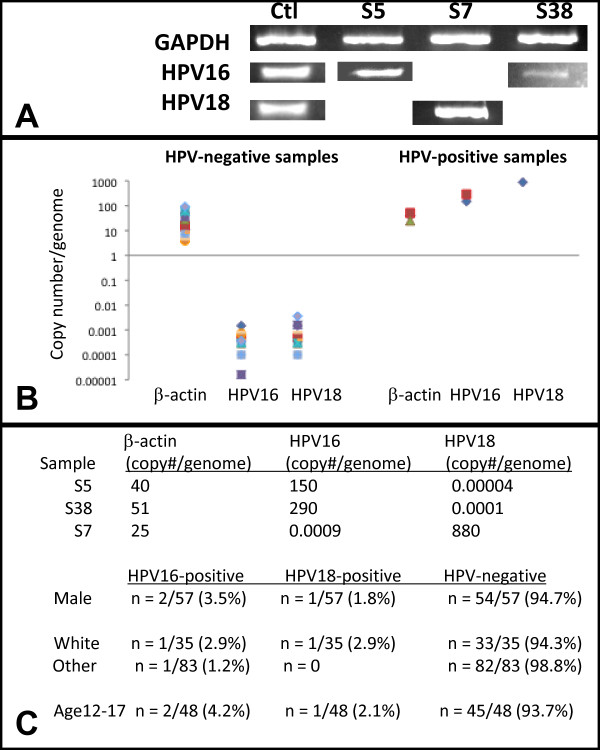
**Saliva sample screening: analysis of qPCR HPV results**. **A**) Three samples were found to harbor HPV DNA: HPV16-positive samples (S5, S38); One sample was HPV-positive (S7). **B**) qPCR analysis revealed HPV-positive samples had values well above the established cutoff value (> 0.001 copies/genome). **C**) All three HPV-positive samples were male, representing 2.5% of the total (n = 118). Two of the three HPV-positive samples were from White participants. All three HPV-positive samples were from the CCSD cohort (ages: 12-17); Both HPV16-positive samples were obtained from 14-year old participants, while the HPV18-positive sample was obtained from a 15-year old.

Samples of extracted DNA were subsequently screened for the presence of HPV16 and HPV18 using PCR (Figure 2). This screening yielded three HPV-positive samples (n = 3/118), representing 2.5% of the total screened. Two of these samples (S5, S38) harbored HPV16 DNA and one was found to harbor HPV18 DNA (S7). Processing of DNA samples using qPCR provided quantitative assessments, as well as measurements of sensitivity and specificity. Analysis of copy number per genome for the housekeeping gene (β-actin) for the HPV-positive (range: 25 - 40 copies/genome) and HPV-negative samples (4 – 93 copies/genome) revealed values that were well above the cutoff value (> 0.1 copies/genome). Results of qPCR analysis revealed copy numbers of HPV-positive samples (range: 150 – 880 copies/genome) that were significantly higher than HPV-negative samples (range: 0.00148 – 0.0000016 copies/genome), which could be distinguished using the cutoff value (> 0.001 copies/genome). These analyses revealed no false positives or false negatives, demonstrating sufficient sensitivity and specificity to ascertain the proportion of true HPV-positive samples (3/118 or 2.5%; 3/3 or 100%) and true HPV-negative samples (115/118 or 97.5%; 115/115 or 100%).

Although the relatively small proportion of HPV-positive samples does not allow for more broad inferences, a descriptive analysis of the demographic information regarding these samples revealed that all three were derived from males (n=3/57 or 5.3%) and none were derived from females. The two HPV16-positive samples were collected from White participants aged 14 (n = 2/35 or 5.7%), while the HPV18-positive sample was collected from a non-White participant aged 15 (n = 1/83 or 1.2%). All three HPV-positive samples were from the CCSD (teenage) cohort (n = 3/48 or 6.25%), while none were observed in the UNLV-SDM (pediatric) cohort (n = 70).

## Discussion

The main goal of this study was to screen normal healthy children and teenagers in Nevada for the presence of high-risk oral HPV. This retrospective analysis used existing saliva samples, collected from pediatric dental patients and local school district teenagers, to obtain novel data from this previously untested juvenile population thereby complementing the ever-growing body of evidence regarding oral HPV prevalence in children. Most importantly, these data have suggested an oral prevalence of high-risk HPV stains of approximately 2.5%.

Recent studies of healthy adults have found similar prevalence rates, ranging between 3.1 and 5% [40,41]. In fact, the recent pilot study of healthy adults in Nevada reported oral HPV in 2.6% of 151 cancer-free patients [42]. The results of the current study were markedly different, however, because two of the three HPV-positive samples were obtained from Whites and all were from males – whereas the prior study of adults found oral HPV among females and minorities only. Other evidence has suggested that although samples from females and males had similar rates of oral HPV (56 and 44%, respectively), the HPV-negative samples from that study were overwhelmingly female (82%) – providing some evidence of a possible male, gender-specific phenomenon similar to the current study results [67]. Although there are many possible explanations, such as the transient nature of the local population, many other factors such as stress, diet, income, poverty, socioeconomic status, and oral HPV exposure may affect different racial groups and both genders in similar ways.

This study represents a significant turning point in public health efforts to elucidate oral HPV detection in a geographic area known for higher than average (and previously increasing) rates of oropharyngeal cancers [43,44]. However, this study had several limitations that must be acknowledged. First, the retrospective nature of this study limited the inferences that could be made, unlike other recent prospective studies of oral HPV in children and adolescents [64-68]. In addition, no detailed behavioral or socioeconomic data were available, such as parental income or smoking behaviors, due to the nature of this retrospective pilot study. It is hoped that future investigations involving oral HPV will provide additional insights with more detailed behavioral information, as well as data regarding housing, education, income and other socioeconomic indicators [67-71].

## Conclusions

This project successfully screened saliva samples for high-risk HPV, confirming both HPV16 and HPV18 strains were present in a small subset. Although previous work has focused on oral HPV transmission from mother to newborn during birth and while nursing [58-63,68], there is growing evidence to suggest that following the perinatal period, oral HPV transmission through close personal contact, such as shared eating utensils, toys, kissing and bathing may account for oral HPV transmission (primarily HPV16) in children and adolescents [72-75]. This study, therefore, provides critical information of significant value to other dental, medical, oral and public health professionals who seek to further an understanding of high-risk HPV prevalence among children as part of a broader understanding of oral health and disease risk in pediatric populations.

## Methods

### Sample size

Given the known prevalence of high-risk oral HPV in the previous study that screened healthy adults within this geographic area, the appropriate sample size was calculated using the following formula: n = *Z*^*2*^*P* (1 – *P*) / *d*^2^, where n = sample size, *Z* = *Z* statistic for a level of confidence, *P* = expected prevalence, and *d* = precision [76,77]. Using the 95% level of confidence, the *Z* value was determined to be 1.96; Using the previous proportion/prevalence of high-risk HPV of 2.6%, the *P* value was set as *P* = 0.026; The value for *d* should be calculated at 0.5 (*P*), resulting in a value for *d* = 0.013. Using these inputs, the minimum sample size for this study was calculated to be n = 75.

### Human subjects

The protocol for this study titled “Retrospective Evaluation of Microbial Presence in Existing Saliva Repository: A PCR-Based Molecular Survey of Oral Microbial Populations from Existing Saliva Samples” was filed, amended, and approved by the UNLV Office of Research Integrity – Human Subjects (OPRS#1104-3801M) on May 10, 2011. The existing saliva samples were collected during two previous studies within the UNLV School of Dental Medicine (SDM) during 2009-2010. Saliva samples collected from a prior study of teenagers (14-15 years old) were derived from a convenience sample of selected schools within the Clark County, Nevada School District (CCSD; n=48); originally obtained for the purpose of surveying levels of oral cariogenic bacteria. Saliva samples collected from young children (2-11 years old) were obtained from a convenience sample within the UNLV-SDM pediatric dental clinic (n=70); originally obtained for the purpose of screening for heavy metal (lead or Pb) burden. In brief, for the original saliva sample collections at CCSD and UNLV-SDM, parents and subjects were recruited onsite. Inclusion criteria: Informed Consent/Parent Permission was required and conducted onsite, as well as an Assent to Participate in Research for all children over the age of seven who were capable of reading. Exclusion criteria: subjects younger than seven years of age, subjects that declined to participate, and subjects with parents who declined to participate. Each sample was assigned a unique, randomly-generated number to prevent research bias, with the only demographic information regarding the saliva specimens: gender, age, and ethnicity of the original study participant. This current project was a retrospective analysis of these two collections of saliva samples; the total combined sample size for this study was n = 118.

### Saliva collection protocol

In brief, subjects who agreed to participate were given a small, sterile saliva collection container, 50 mL sterile polypropylene tube (Fisher Scientific: Fair Lawn, New Jersey, USA). Participants were then asked to chew on a small piece of paraffin wax for one minute and then to expectorate, in accordance with the previous pilot study protocol [42]; sample volumes varied from approximately 50 μL to 2.5 μL. Samples were stored on ice until transport to a biomedical laboratory for analysis. Each saliva sample was assigned a unique, randomly-generated number to prevent research bias. Demographic information regarding the sample was concurrently collected, which consisted of age, gender, and ethnicity only.

### Cell counting and DNA isolation

All samples were centrifuged for 10 minutes at 2,100 g (RCF) and the cell pellet washed with 1X phosphate-buffered saline (PBS) (HyClone: Logan, Utah, USA) and resuspended in 5 mL of 1X PBS. Cell number was determined using Trypan Blue (Fisher Scientific: Fair Lawn, New Jersey, USA) using a Zeiss Axiovert 40 inverted microscope (Carl Ziess, Inc: Thornwood, New York, USA) and a hemacytometer (Fisher Scientific: Fair Lawn, New Jersey, USA). To determine if any samples harbored the HPV virus, DNA was isolated from the saliva sample using a minimum of 3.5 x 10^5^ cells using the GenomicPrep DNA isolation kit (Amersham Biosciences: Buckinghamshire, United Kingdom), using the procedure recommended by the manufacturer as previously described [12,42,78,79]. DNA purity was calculated using ratio measurements of absorbance at 260 and 280 nm (A260/A280 ratio between 1.7 and 2.0).

### Polymerase chain reaction (PCR)

DNA from each sample was then used to perform PCR with the Fisher exACTGene complete PCR kit (Fisher Scientific: Fair Lawn, New Jersey, USA) and a Mastercycler gradient thermocycler (Eppendorf: Hamburg, Germany) using the following primers for HPV16 [12,78], HPV18 [12,78,79], and glyceraldehyde- 3- phosphate dehydrogenase (GAPDH) [80] (SeqWright: Houston, Texas, USA):

HPV16 forward primer, ATGTTTCAGGACCCACAGGA;

HPV16 reverse primer, CCTCACGTCGCAGTAACTGT.

HPV18 forward primer, ATGGCGCGCTTTGAGGATCC;

HPV18 reverse primer, GCATGCGGTATACTGTCTCT;

GAPDH forward primer, ATCTTCCAGGAGCGAGATCC;

GAPDH reverse primer, ACCACTGACACGTTGGCAGT;

One μg of template DNA was used for each reaction. The initial denaturation step ran for three minutes at 94°C. A total of 30 amplification cycles were run, consisting of 30 second denaturation at 94°C, 60 seconds of annealing at 58°C, and 30 seconds of extension at 72°C. Final extension was run for five minutes at 72°C. The PCR reaction products were separated by gel electrophoresis using Reliant 4% NuSieve® 3:1 Plus Agarose gels (Lonza: Rockland, Maine, USA). Bands were visualized by UV illumination of ethidium-bromide-stained gels and captured using a Kodak Gel Logic 100 Imaging System and 1D Image Analysis Software (Eastman Kodak: Rochester, New York, USA).

### Quantitative PCR (qPCR)

DNA samples were then processed using qPCR to provide more specific and sensitive quantification. Primers and probes were designed using Roche Universal Probe library (UPL) assay design software to amplify the region overlapping E6 and E7 gene sequence of HPV16 (GenBank accession no. K02718) and the human β-actin housekeeping gene (GenBank accession no. M10277). These primers were purchased from Sigma-Aldrich (St. Louis, Missouri, USA) and probes from Roche Applied Science (Indianapolis, Indiana, USA).

HPV16 E6/E7 forward primer 5′-CAACTGATCTCTACTGTTATGAGCAA-3′, HPV16 E6/E7 reverse primer 5′-CCAGCTGGACCATCTATTTCA-3′, HPV16 E6/E7 hydrolysis “Taqman” probe 5′-(fam)-AGGAGGAG-(dark quencher dye)-3′ (UPL probe #63) was used to amplify the 73 base pair (bp) region between the 535 nucelotide (nt) position and 607 nt position. HPV18 E7 forward primer 5′-GACTCAGAGGAAGGAAAACGATGAAA, HPV18 E7 reverse primer 5′-GTGACGTTGTGGTTCGGCT; HPV18 E7 probe 5′-TGGAGTTAATCATCAACATTTACCA was used to amplify the 25 bp region between the 715 and 739 nt position. Human β-actin forward primer 5′-GTGGGGTCCTGTGGTGTG-3′, human β-actin 5′-GAAGGGGACAGGCAGTGA-3′, human β-actin hydrolysis “Taqman” probe 5′-(fam)-GGGAGCTG-(dark quencher dye)-3′ (UPL probe #24) amplified the 61 bp region between 2642 nt position and 2702 nt position.

The real-time reaction mixture was prepared in a LightCycler® 480 multiwell Plate 96 containing 1x LightCycler® 480 Probes Master (Roche Applied Sciences: Indianapolis, Indiana, USA), 1 μM of each respective primer set (forward and reverse), 0.2 μM of respective probe, and 2 μl of DNA template; in a 20 μl final reaction volume. The probes master mix contained reaction buffer, dNTP mix (including dUTP in place of dTTP), 3.2 mM MgCl_2_, and *Taq* DNA polymerase. The real-time PCR assay was performed on a LightCycler 480 system (Roche Applied Sciences: Indianapolis, Indiana, USA) with the following cycle parameters: pre-incubation for initial enzyme activation at 95°C for 10 minutes, followed by 45 cycles of 95°C for 10 seconds (ramp rate 4.4°C/second), 60°C for 30 seconds (ramp rate 2.2°C/second) and 72°C for 1 second (ramp rate 4.4°C/second). Following amplification phase, a cooling step was performed at 40°C for 30 seconds (ramp rate of 2.2°C/ second). Acquisition of the fluorescence signal was performed using Mono Hydrolysis Probe setting (465-510 nm) following the 72°C extension phase of each cycle. All samples were carried out in triplicate.

The CaSki (American Type Culture Collection; Manassas, Virginia, USA) cervical adenocarcinoma cell line was used to develop standard curves for both the HPV16 (600 copies/genome) and GAPDH (2 copies/genome) genes. The GH354 (American Type Culture Collection; Manassas, Virginia, USA) cervical adenocarcinoma cell line was used to develop the standard curves for HPV18 (200 copies/genome). DNA extracted from CaSki and GH354 cells were serially diluted tenfold starting at 50 ng to 0.0005 ng [81] Quantification was achieved using Cycle Threshold (C_T_) measured with the second derivative maximum method (LightCycler 480 Software version 1.5.0.39; Roche Applied Sciences: Indianapolis, Indiana, USA). Saliva samples > 0.001 copy/genome were considered HPV positive. Specificity analysis was performed on qPCR assay against HPV18 and found to be 100% specific (data not shown).

### Statistical evaluation

Sensitivity and specificity were calculated as the proportion of true positives and true negatives (cutoff value >0.001 copies/genome), respectively. Following the acquisition of saliva samples and HPV screening results, demographic information from the samples were compared with the overall demographic profile of the local population using a chi-square (χ2) test, to determine if any characteristic (gender, race, age) was different than expected among the subjects evaluated in this study (n = 118). A probability level of alpha (α) = 0.05 was used to determine statistical significance.

## Abbreviations

HPV: Human papillomavirus; DNA: Deoxyribonucleic acid; US: United States; PCR: Polymerase chain reaction; OPRS: Office for the Protection of Human Research Subjects; CCSD: Clark County Nevada - School District; UNLV-SDM: University of Nevada Las Vegas - School of Dental Medicine; RCF: Relative centrifugal force; PBS: Phosphate-buffered saline; GAPDH: Glyceraldehyde-3- phosphate dehydrogenase; qPCR: Quantitative polymerase chain reaction; bp: Base pair; nt: Nucleotide; dNTP: Deoxyribonucleotide triphosphate; dUTP: 2-Deoxyuridine triphosphate; dTTP: Deoxythymidine triphosphate.

## Competing interests

The authors declare they have no competing interests.

## Authors’ contributions

KK and CF conceived, monitored, and coordinated the experimental design. KH, EE, JA and AH were responsible for recruiting subjects, informed consent, collecting samples, and some biomedical analysis. CF, EE, JA, and AH carried out the DNA extractions, PCR, and qPCR analysis. KK and KH were responsible for the data analysis, as well as the writing and editing of this manuscript. All authors read and approved the final manuscript.

## Pre-publication history

The pre-publication history for this paper can be accessed here:

http://www.biomedcentral.com/1472-6831/12/43/prepub
